# Prevalence of Abuse, Perceived Stress, and Resilience Among Tribal Girls: A Comparison With Girls Attending Government Schools

**DOI:** 10.7759/cureus.105483

**Published:** 2026-03-19

**Authors:** Anjali Sharma, Dhruvi Jeenger, Shivangi Desai, Chhayank H Acharya

**Affiliations:** 1 Psychiatry, Geetanjali Medical College and Hospital, Udaipur, IND; 2 Mathematics and Statistics, Rockwoods International School, Udaipur, IND

**Keywords:** child abuse, india, perceived stress, resilience, tribal girls

## Abstract

Background: Tribal girls constitute a vulnerable population due to gender and socioeconomic disadvantage and limited access to education and health services. They are frequently exposed to adverse experiences that cause psychological distress and increase their risk of psychopathology.

Aim: To assess and compare the prevalence of abuse, perceived stress, and resilience among tribal and government school-going girls.

Methodology: A cross-sectional comparative study was conducted among 581 adolescents, including 298 tribal girls attending non-governmental organization (NGO) run tribal residential schools (TRS) and 283 adolescent girls attending urban government schools, between the age group of 12 and 17 years. Abuse, perceived stress, and resilience among participants were assessed using the Hindi-validated versions of the International Society for the Prevention of Child Abuse and Neglect (ISPCAN) Child Abuse Screening Tool - Children’s Version (ICAST-C), the Perceived Stress Scale (PSS-10), and the Child and Youth Resilience Measure (CYRM-R), respectively. Group differences were analyzed using the chi-square test and the independent t-test, and Pearson’s correlation was used to examine associations between abuse, stress, and resilience.

Results: Tribal girls reported higher levels of emotional and sexual abuse and perceived stress (*P *< 0.001). Resilience scores were higher among tribal girls (*P *< 0.001), possibly reflecting stronger coping mechanisms and bonding among peers within the structured environment of residential schools. Higher levels of abuse were linked to higher levels of stress in both groups (*P *< 0.001). For tribal girls, abuse and stress were negatively correlated with resilience (*P *< 0.001).

Conclusions: Emotional and sexual abuse, perceived stress levels, and resilience are significantly greater among tribal adolescent girls. These vulnerable adolescents need early intervention to reduce the psychological effects of adverse childhood experiences.

## Introduction

One in four school-going adolescents in India has a mental health issue, with girls often bearing a higher burden of symptoms [1]. Scheduled Tribes (STs) are constitutionally recognized indigenous communities in India characterized by socioeconomic disadvantage, geographic isolation, and distinct cultural practices. Tribal girls are female adolescents belonging to these communities. ST constitute nearly 9% of India’s population, about 104 million people, and their growth rate (23.7%) is higher than the national growth rate (17.6%) [2]. A substantial proportion of adolescents living in underserved urban areas constitutes a high-risk group [2,3]. Tribal girls face added disadvantages such as poverty, remoteness, early marriage, and limited access to health care and education, which increase their vulnerability to abuse and chronic stress [4]. Perceived stress refers to the degree to which individuals appraise situations in their lives as unpredictable, uncontrollable, and overwhelming, reflecting subjective psychological stress rather than objective life events [5]. Tribal adolescent girls have undernutrition, anaemia, and poor access to healthcare, further compromising their well-being [4]. Indian research indicates that tribal girls experience greater psychological distress than non-tribal peers, linked to gender disadvantage, violence, and limited autonomy [6,7].

Child abuse consists of exploitation that results in actual or potential harm to a child’s health, development, or dignity. It includes physical abuse, emotional abuse, sexual abuse, neglect, and exposure to domestic violence [8]. Tribal adolescent girls are at greater risk of abuse and maltreatment due to traditional patriarchal norms, restrictive gender roles, and limited access to education and support services [9,10]. Kumar et al. found that 97.3% of school-going adolescents in a Kerala-based study reported at least one form of abuse, including physical, emotional, and sexual forms [11]. Indian studies conducted in general school-going populations show high rates of maltreatment, with girls disproportionately affected, especially in cases of sexual abuse and neglect [9,10]. A community-based study in urban Jharkhand among tribal adolescent girls found that 42.7% had experienced some form of abuse or maltreatment [4].

Exposure to any form of abuse in adolescence creates chronic stress, which, in turn, heightens long-term psychological vulnerability [12]. Tribal and urban Indian girls experience higher stress levels due to socioeconomic disadvantage [6,12,13]. A study in central India reported moderate to severe psychological distress in 27.5% of tribal girls [6], while research from Jharkhand and Kerala showed high stress among tribal women compared to non-tribal groups [12,13].

Resilience is defined by the ability to respond positively and thrive in response to adverse situations [14]. It reflects a dynamic adaptive response to challenge, rather than mere absence of problems, enabling adolescents to adjust successfully despite risks [15,16]. Researchers have identified that a lack of resilience in children and adolescents may lead to psychosocial maladaptation and psychopathology in adulthood [14]. Low resilience was reported in 62.3% of urban adolescents in a Kolkata school-based study and in 51% of tribal girls in a study conducted at Chhattisgarh [6,17].

Urban government schools cater to a large proportion of adolescents who are from the same regional and socioeconomic context but without tribal residential schooling exposure. Also, urban government school girls live within their family environments, allowing comparison across differing cultural and caregiving contexts. Studies among urban school-going girls in India report abuse prevalence ranging from 50% to over 90%, depending on measurement tools and definitions [11,10]. Similarly, moderate to high perceived stress has been observed among 30%-60% of urban adolescent girls in school-based studies [18]. Based on existing literature suggesting higher vulnerability among tribal adolescents, we hypothesized that tribal girls would report higher levels of abuse and perceived stress compared to government school girls, and that abuse would be positively associated with stress and negatively associated with resilience.

There is a lack of Indian published studies that assess multiple parameters and provide a head-to-head comparison between tribal adolescent girls living in a hostel and urban government school-going girls living with parents. Hence, this study aimed to assess the prevalence of abuse and maltreatment, perceived stress, and resilience of tribal girls and compare them with those of government school girls.

## Materials and methods

This cross-sectional comparative study was conducted among adolescent girls from tribal communities and urban government schools. Before commencing the study, approval was obtained from the Institutional Research Review Committee (IRRC) (GMCH/IRRC/PG24/2025/3240) and the Institutional Ethics Committee (IEC) (GU/HREC/EC/2025/2931). This study was conducted from September 2025 to November 2025.

Study population and sampling

There were two groups in the present study: one group comprising tribal girls, who were recruited from residential tribal schools, where they reside in hostel settings away from their families, and another group comprising urban government school-going girls representing non-tribal adolescents living with their parents in urban settings. There are 14 girls' tribal residential schools (TRS) in this division and a total of 17 government girls’ senior secondary schools in Udaipur [19]. The sample size was calculated using the following formula, taking the prevalence of any abuse in tribal adolescent girls as ~ 43%, based on a community-based study from urban Jharkhand [4]. The required sample size was 262 girls per group. Thus, the minimum total sample size was 524 adolescent girls. We aimed to recruit a total of nearly 580 participants, allowing for 10% non-responses.

The sample size was calculated using the formula:

\[ n = \frac{Z_{\alpha/2}^{2} \, p(1 - p)}{d^{2}} \]

Here, *n* is the required sample size, *Z* is the standard normal deviation for the desired confidence level (1.96 for 95% confidence interval), *p* is the prevalence, and *d* is the absolute precision.

A multistage random sampling technique was employed for the study. We randomly selected three TRS girls’ schools and three urban government schools from Udaipur. From the selected schools, students were selected from each eligible class using computer-generated random numbers to have equal representation. Participation was voluntary, and response rates were high. A total of 298 eligible adolescent girls in the community school participated in the study and were compared with 283 adolescent girls from three urban government schools in the division. Before data collection among TRS adolescent girls, formal permission 2 of 10 was obtained from the President of the TRS and assent from the adolescent girls. For Urban Government Schools, formal permission from the respective school principals, written informed consent from parents, and assent from adolescent girls were obtained.

Inclusion and exclusion criteria

The inclusion criteria comprised adolescent girls aged 12-17 years. For the tribal group, participants were girls enrolled in TRS during the study period. For the comparison group, participants were adolescent girls enrolled in selected urban government schools and residing with their parents. All participants were required to provide assent, and written informed consent was obtained from their parents or legal guardians before enrollment. Adolescents with known physical or intellectual disabilities were excluded from the study.

Procedure

A semi-structured sociodemographic proforma was designed to collect data. The tools used in the present study were self-administered in the Hindi language. On the days of data collection, the investigators visited the TRS and government schools, and they addressed any queries raised by students. The International Society for the Prevention of Child Abuse and Neglect (ISPCAN) Child Abuse Screening Tool - Children’s Version (ICAST-C) was used to assess all forms of abusive experiences [8]. The Perceived Stress Scale (PSS-10) was used to assess the level of stress, and resilience was assessed using the Child and Youth Resilience Measure (CYRM-R) [5,20]. Partially filled or incomplete questionnaires were excluded from analysis, and completed questionnaires and proformas were collected during the collection period. During data collection, adequate physical spacing between participants was ensured to protect children’s privacy, and no identifying information was collected. The children were informed that if any immediate safety concerns were reported, the warden or principal would be notified.

Tools used

The International Society for the Prevention of Child Abuse and Neglect (ISPCAN) Child Abuse Screening Tool Children’s Version (ICAST-C)

The ICAST-C is a standardized self-report screening instrument developed by ISPCAN to assess lifetime experiences of child maltreatment. The tool has been widely used in international and Indian studies and is suitable for use among school-going children and adolescents, with acceptable internal consistency, with Cronbach’s alpha coefficients ranging from 0.61 to 0.82, and good cross-cultural validity. It consists of 38 total items, of which 35 items contribute to the five abuse/violence domains used for subscale scoring: violence exposure (seven items), physical victimization (nine items), psychological victimization (seven items), sexual victimization (six items), and neglect (six items). Using the three-point scoring method (0-2 per item), the minimum possible score for each domain is 0, and the maximum score equals 2 multiplied by the number of items in that domain (violence exposure 0-14, physical 0-18, psychological 0-14, sexual 0-12, neglect 0-12). The summed total score across the 35 abuse/violence items, therefore, ranges from 0 to 70. With this method, response categories were collapsed as 0 = no abuse experience (*never happened*) and 1 or 2 = abuse present/occurred [8,11].

The Perceived Stress Scale (PSS-10)

This is a 10-item instrument scored on a 5-point Likert scale ranging from 0 (never) to 4 (very often), developed by Cohen, Kamarck, and Mermelstein in 1983 to measure the degree to which individuals appraise situations in their lives as stressful [5]. The Hindi version of the PSS-10 has demonstrated acceptable internal consistency (Cronbach’s alpha ≈ 0.74) and construct validity in Indian samples, including adolescent school students, with Cronbach’s alpha around 0.74 [18,21]. PSS-10 scores range from 0 to 40; 0-13 = low stress, 14-26 = moderate stress, and 27-40 = high stress [22].

The Child and Youth Resilience Measure - Revised (CYRM-R)

The CYRM-R [20] was developed by the Resilience Research Centre as a self-report tool for youth (ages 10-23 years) to assess social-ecological resources for resilience. It comprises 17 items divided into two subscales: personal and caregiver. Each item is rated on a 5-point Likert scale ranging from 1 to 5, with higher scores indicating greater resilience. The Hindi version of the CYRM has been validated in Indian school-going adolescents, demonstrating good internal consistency, with Cronbach’s alpha of about 0.82 [23]. The highest and lowest attainable scores are 17 and 85, respectively, and the median is 62. In the absence of established normative cutoff values for Indian adolescents, resilience was dichotomized using the sample median score (62) for analytical purposes, consistent with prior Indian school-based study; scores above *62 *were categorized as higher resilience, and scores ≤62 as lower resilience [17].

Statistical analysis

The data were entered into an Excel Sheet, and statistical analysis was carried out using the SPSS software version 16 (SPSS Inc., Chicago, IL). Data distribution was assessed for normality using the Shapiro-Wilk test. The chi-square test was used to compare the differences in the proportions between the groups. Homogeneity of variance was evaluated using Levene’s test. An unpaired Student's t-test was used to compare the mean and standard deviation of both groups; Pearson’s correlation was calculated between the total scores of ICAST, PSS, and CYRM-R. All statistical analyses were done at a 95% confidence interval, and *P* < 0.05 was considered statistically significant.

## Results

A total of 581 adolescent girls participated in the study, including 298 (51.3%) tribal girls and 283 (48.7%) girls from urban government schools. The two groups were comparable in age distribution. More than half of the girls in both groups were studying in Classes 8th-10th, and nearly three-fourths were first- or second-born children. Nuclear families were more common among government school girls (183, 64.7%) than tribal girls (168, 56.4%), whereas joint families were relatively more frequent among tribal girls. A higher proportion of tribal girls reported single-parent families, parental substance use, parental unemployment, and domestic violence at home compared to their government school counterparts (Table 1).

**Table 1:** Sociodemographic characteristics of tribal and government school girls. N = number of girls. Values are shown as *n* (%) for each column. *Not known* indicates unreported or missing information. *P* < 0.05 was considered statistically significant.

Sociodemographic variables	Tribal girls, *N* = 298	Government school girls, *N *= 283	Chi-square	*P*-value
	*n* (%)	*n* (%)		
Age (in years)				
12	30 (10.1%)	20 (7.1%)	2.81	0.73
13	50 (16.8%)	45 (15.9%)		
14	60 (20.1%)	55 (19.4%)		
15	60 (20.1%)	65 (23.0%)		
16	50 (16.8%)	55 (19.4%)		
17	48 (16.1%)	43 (15.2%)		
Education status				
Class 6th	40 (13.4%)	30 (10.6%)	4.3	0.507
Class 7th	55 (18.5%)	50 (17.7%)		
Class 8th	70 (23.5%)	60 (21.2%)		
Class 9th	65 (21.8%)	60 (21.2%)		
Class 10th	45 (15.1%)	50 (17.7%)		
Class 11th	23 (7.7%)	33 (11.7%)		
Birth order				
1st	120 (40.3%)	110 (38.9%)	0.41	0.938
2nd	103 (34.5%)	105 (37%)		
3rd	55 (18.5%)	50 (17.7%)		
4th or more	20 (6.7%)	18 (6.4%)		
Family type				
Nuclear	168 (56.4%)	183 (64.7%)	3.83	0.05
Joint	130 (43.6%)	100 (35.3%)		
Number of close friends				
None	15 (5.0%)	10 (3.5%)	6.99	0.072
One	80 (26.8%)	60 (21.2%)		
Two to Three	150 (50.3%)	140 (49.5%)		
Four or More	53 (17.8%)	73 (25.8%)		
Parental status				
Single	80 (26.8%)	60 (21.2%)	2.23	0.135
Together	218 (73.2%)	223 (78.8%)		
Parental substance use				
Yes	120 (40.3%)	90 (31.8%)	4.15	0.042
No	178 (59.7%)	193 (68.2%)		
Domestic violence				
Yes	70 (23.5%)	45 (15.9%)	4.8	0.028
No	228 (76.5%)	238 (84.1%)		
Parental employment				
Both employed	80 (26.8%)	110 (38.9%)	12.94	0.005
One employed	150 (50.3%)	130 (45.9%)		
Both unemployed	40 (13.4%)	20 (7.1%)		
Not known	28 (9.4%)	23 (8.1%)		

Table 2 shows that tribal girls reported significantly higher emotional abuse, sexual abuse, total ICAST-C scores, and higher perceived stress levels (*P *< 0.001) than government school girls. Tribal girls reported significantly higher personal, caregiver, and total resilience scores (*P *< 0.001).

**Table 2:** Comparison of child abuse and maltreatment, perceived stress, and resilience between tribal girls and government school girls. Values are mean ± standard deviation. Group differences were tested using independent-samples t-tests. *P < 0.05 was considered statistically significant. ISPCAN, International Society for the Prevention of Child Abuse and Neglect; ICAST-C, ISPCAN Child Abuse Screening Tool - Children’s Version; PSS-10, 10-item Perceived Stress Scale; CYRM-R, Child and Youth Resilience Measure - Revised

Domains	Tribal girls	Government school girls	*t*-test	*P*-value
	(*N* = 298)	(N = 283)		
	Mean ± SD	Mean ± SD		
ICAST-C				
Physical abuse	9.2 ± 4.0	9.0 ± 3.8	0.62	0.537
Emotional abuse	8.6 ± 3.4	7.4 ± 3.2	4.38	<0.001*
Sexual abuse	5.8 ± 2.1	4.9 ± 1.9	5.42	<0.001*
Neglect	3.1 ± 1.8	2.9 ± 1.7	1.38	0.168
Witnessing violence	3.7 ± 3.0	3.4 ± 2.8	1.25	0.213
Total score	30.5 ± 6.0	27.9 ± 5.6	5.4	<0.001*
PSS-10				
PSS score	19.1 ± 6.3	17.0 ± 6.0	4.12	<0.001*
CYRM-R				
Personal resilience	37.5 ± 5.6	35.7 ± 5.4	3.94	<0.001*
Caregiver resilience	26.0 ± 4.5	24.4 ± 4.3	4.38	<0.001*
Total resilience	63.5 ± 8.1	60.1 ± 7.7	5.19	<0.001*

Table 3 shows 70.5% of tribal girls had experienced significantly higher abusive experiences than their government school counterparts (*P* = 0.008). Higher stress levels are more prevalent among tribal girls (*P *= 0.011). In contrast, tribal girls had significantly higher resilience compared to government school girls (*P *< 0.001).

**Table 3:** Distribution of abuse and maltreatment (ICAST-C), perceived stress (PSS-10), and resilience (CYRM-R) between tribal girls and government school girls. Values are shown as *n* (%). Group differences were tested using the chi-square test. Abuse present = reporting ≥1 physical, emotional, or sexual ICAST-C item at any frequency. Resilience was dichotomized at ≤62 vs. >62 on the CYRM-R. *P < 0.05 was considered statistically significant. ISPCAN, International Society for the Prevention of Child Abuse and Neglect; ICAST-C, ISPCAN Child Abuse Screening Tool - Children’s Version; PSS-10, 10-item Perceived Stress Scale; CYRM-R, Child and Youth Resilience Measure - Revised

Variables	Tribal girls (*N* = 298)	Government school girls (*N* = 283)	Chi-square	*P*-value
	*n *(%)	*n* (%)		
Abuse and maltreatment				
Present	210 (70.5%)	170 (60.1%)	6.94	0.008*
(Physical, emotional, or sexual)				
Perceived stress				
Low stress (0-13)	60 (20.1%)	85 (30.0%)	9.07	0.011*
Moderate stress (14-26)	194 (65.1%)	170 (60.1%)		
High stress (27-40)	44 (14.8%)	28 (9.9%)		
Resilience				
Low resilience scores (<=62)	125 (41.9%)	164 (58.0%)	14.87	<0.001*
High resilience (>62)	173 (58.1%)	119 (42.0%)		

In both groups, higher total ICAST-C scores were significantly associated with higher perceived stress levels (*P *< 0.001). Among tribal girls, abusive experiences and stress had a significant negative correlation with total resilience scores (*P *< 0.001) (Table 4).

**Table 4:** Correlation between abuse and maltreatment (ICAST-C), perceived stress (PSS-10), and resilience (CYRM-R) among tribal girls and government school girls. Values are Pearson’s correlation coefficients (*r*). Tribal girls (*N* = 298); government school girls (*N* = 283). *P < 0.05 was considered statistically significant. ISPCAN, International Society for the Prevention of Child Abuse and Neglect; ICAST-C, ISPCAN Child Abuse Screening Tool - Children’s Version; PSS-10, 10-item Perceived Stress Scale; CYRM-R, Child and Youth Resilience Measure - Revised

Domains	Groups	ICAST-C, *r* (*p*)	PSS-10, *r* (*p*)	CYRM-R, *r* (*p*)
ICAST-C Total scores	Tribal girls	1	0.380 (<0.001*)	-0.210 (<0.001*)
	Government school girls	1	0.320 (<0.001*)	-0.070 (0.240)
PSS-10 scores	Tribal girls	0.380 (<0.001*)	1	-0.290 (<0.001*)
	Government school girls	0.320 (<0.001*)	1	-0.090 (0.140)
CYRM-R scores	Tribal girls	-0.210 (<0.001*)	-0.290 (<0.001*)	1
	Government school girls	-0.070 (0.240)	-0.090 (0.140)	1

## Discussion

Community-based evidence from rural India highlights notable mental health problems faced by adolescent girls around well-being, stress, and psychosocial difficulties [4]. STs continue to face long-standing socioeconomic disadvantages such as poverty, gaps in education and healthcare access, and marginalization, which can contribute to a higher burden of stress and mental health problems [1,2]. Research from a tribal area in central India establishes a connection between gender disadvantage and psychological distress in adolescent girls, underscoring the importance of social circumstances [6]. The cumulative disadvantage related to gender and geographic marginalization places tribal girls at heightened risk for internalizing mental health problems [24]. This study aimed to assess abuse, perceived stress, and resilience among TRS girls and compare them with girls attending urban government schools.

The present study found that nuclear families were more common in both groups; however, the proportion of nuclear families was lower among tribal girls compared to government school girls, with relatively higher representation of joint families among tribal adolescents, aligning with findings of Basu et al. [25]. Our study observed that 40.3%, 23.5%, and 13.4% tribal adolescents were exposed to parental substance use, domestic violence, and parental unemployment, respectively. Shrivastav et al. reported similar levels of family conflict and parental substance use among adolescents in Central rural India [6]. Although joint families offer support, they can also expose adolescent girls in disadvantaged tribal settings to intergenerational conflicts, unemployment, and domestic violence, contributing to long-term mental health vulnerability [26]. These findings together underline the significance of family structure and environment in adolescent girls’ well-being.

Charak and Kumar et al. showed that tribal girls experience greater levels of emotional and sexual abuse compared to girls in government schools [9,11]. Research from central India, Jharkhand, and Kerala indicated that tribal females experience increased stress levels and worse quality of life compared to government school girls [6,12,13]. The present study found similar results. Research shows higher resilience among Paniya tribal adolescents [27]. Our findings aligned with this, revealing higher levels of personal, caregiver, and overall resilience in tribal adolescent girls. Research shows higher resilience among Paniya tribal adolescents [27]. Our findings aligned with this, revealing higher levels of personal, caregiver, and overall resilience in tribal adolescent girls. However, structured daily routines, peer cohesion, shared living experiences, and continuous adult supervision in hostel settings may foster adaptive coping and social support mechanisms [28]. This reflects that sustained exposure to adversity might strengthen adaptive coping and reliance on community support groups, despite higher psychosocial risks.

Indian literature also notes disproportionately higher maltreatment exposure among girls, particularly for sexual abuse and neglect [9,10]. Similarly, our study observed that 70.5% of tribal girls experienced abuse and maltreatment, significantly higher than their government school counterparts. Edlina et al. found that girls from tribal communities showed about 60% resilience, which is similar to the 58.1% of high resilience shown by tribal adolescent girls in our study [29]. Similar findings were observed in a Kerala-based study, whereas a Kolkata-based study reported low resilience among tribal adolescents [17,27]. The differences in study population, sampling methods, and measurement tools substantially influence the observed resilience levels.

The present study found that abuse and maltreatment in both tribal and government school girls were strongly correlated with higher perceived stress levels. This aligns with previous Indian literature, especially within poorer and tribal communities [11,12,13]. A study conducted in South India reported a significant negative association between childhood abuse exposure and overall resilience, aligning with our findings [30]. Additionally, the strong negative correlation between perceived stress levels and resilience found in our study aligns with findings from a Mumbai-based study, which indicated that higher stress in adolescent girls is associated with lower resilience. Resilience may buffer the psychological impact of abuse through social and caregiver support, underscoring the need for supportive policies and community-based approaches for vulnerable girls [28].

Strengths and limitations

This study offers a multidimensional assessment of abuse, perceived stress, and resilience among tribal and urban government school-going girls, using validated and reliable self-administered tools in a substantial sample size. Our study has certain limitations. The cross-sectional design restricts causal inference, and reliance on self-administered questionnaires may have introduced recall and social desirability bias, particularly regarding abuse. Given the sensitive nature of abuse reporting within a school setting, fear of disclosure and cultural stigma may also have contributed to underreporting. Adolescents with physical or intellectual disabilities were excluded, which may introduce selection bias and potentially underestimate the overall psychosocial burden. This study did not include adolescent boys, private-school students and participants were drawn from only one region; generalizability is therefore limited. Differences in living arrangements between groups and the lack of adjustment for potential confounders such as socioeconomic status, parental education, and parental mental health may have influenced the observed associations. Further studies should be multicentric with more diverse samples and use longitudinal study designs.

The high prevalence of abuse and perceived stress among tribal adolescent girls highlights the urgent need for school-based screening and early identification within TRS. Given the association between abuse and stress, strengthening child protection mechanisms, sensitizing school staff, and establishing confidential reporting systems are essential. Peer support systems and community-based protective factors should be enhanced by group-based psychosocial interventions. Policymakers should prioritize school mental health services and integrate psychosocial support within educational and tribal welfare programs.

## Conclusions

Tribal girls had significantly greater emotional and sexual abuse as compared to government school girls. They also had higher exposure to all forms of abuse and maltreatment, elevated perceived stress, and higher resilience compared to their urban government school-going counterparts. Among tribal girls, abuse and maltreatment were significantly associated with resilience. These findings highlight the vulnerability of tribal girls and the psychological impact of abuse and maltreatment in this vulnerable group. Policy makers should have increased attention to these vulnerable adolescent girls, and there is a need for early identification and intervention to reduce the psychological and developmental effects of adverse childhood experiences. Future investigations should examine the longitudinal impact of such interventions on stress reduction and resilience enhancement.
